# Surgery

**Published:** 1837-10

**Authors:** 


### SURGERY.

*An Inquiry into the Possibility of Transplanting the Cornea, with a view of relieving blindness (hitherto deemed incurable,) caused by several Diseases of that Structure.* By S. L. L. Bigger, m.b. &c.

The title of this paper will startle those who are not familiar with the feats performed by modern ophthalmologists for the relief of imperfections of the organ of vision. The paper itself, however, may probably be referred to hereafter, in a more advanced stage of ocular surgery, as a record of an early attempt at effecting a bold design, eventually rendered successful by the improvements of time. The operation contemplated by Dr. Bigger, that of "excising the morbid cornea, and replacing it by a healthy structure, taken from some of the inferior animals," is not an original one, having been previously attempted by several surgeons in Germany, on the inferior animals, and with partial success. Dr. Bigger has followed up the practice to a much greater extent, and, from an improved method, with more success than his predecessors. Still the results of his experiments prove that much is yet wanting to render the operation one that can be relied on with much confidence. We think, however, that Dr. B. deserves the greatest credit for his bold and ingenious attempts to remedy one of the most lamentable of ailments. No attempt has yet been made to extend the operation to the human eye. The following extracts from the Report of Dr. Bigger's paper contain (1,) an account of the manner of performing this curious operation; (2,) the results of the operator's last experiments; and (3,) Dr. B.'s conjectures respecting the applicability of the operation to the human eye.

1. "Having fixed, with a ligature, the upper eyelid of the animal from which the cornea is to be taken, he introduces Beer's cataract knife (holding it horizontally, and at first directing it a little backwards, so as to ensure its passing through all the layers of the cornea,) with its edge turned upwards, into that part of the cornea situated about a line or more from its most inferior junction with the sclerotic, and about the same distance external to the mesial line of the eye. He then pushes on the knife for the space of one or two lines, inclining the handle, so that the point of the knife may be brought forward, and caused to pierce the cornea again, at a distance, as small as possible from the point of entrance. The knife should now be pushed on, when it will make as large a section as may be required, which being turned down, is to be cut off with a pair of scissors. The eyelids are then to be closed, to prevent the escape of the crystalline lens and vitreous humour. The excised cornea should be placed on a slip of cork, and the curved needles, carrying very fine ligatures, (two, three, or four in number,) should be passed through the cornea and the piece of cork. The latter, which has been chiefly used as a support to enable the operator to pass the needles through the tough layers of the cornea, should then be broken off, and the cut surfaces of the cornea should be kept moistened with some of the secretion from the eye. The surgeon then proceeds to perform the same operation on the eye to which the cornea is intended to be transplanted. Having done this, and closed the lids for a few moments, until the spasmodic action of the muscles of the eye diminishes, the operator proceeds to adapt the cornea to its new situation, and for this purpose inserts the point of his needle carefully between the margin of the now prolapsed iris and the remains of the cornea, and pressing externally with the nail of the other forefinger against the point of the needle, so as to make it pass through the cornea without dragging or injuring the eye, draws out the needle. To accomplish the latter object, Dr. Bigger was often obliged to use a small forceps, and in this case, the thumb and finger nails of the other hand must be pressed closely and firmly against the cornea on either side of the needle, to obviate any injurious disturbance or dragging of the eye. The ligatures should then be carefully tied, and the ends cut off. Dr. Bigger has found two ligatures to answer the purpose quite as well as four. Finally, the operator clears away any lymph or blood which may have collected on the eye, and concludes the operation by smearing the eyelids with a little spermaceti ointment."

2. "On his return to Dublin, Dr. Bigger commenced his experiments anew; of these, he has now performed eighteen. The subjects of the first and last, two rabbits, were presented before an evening meeting of the King and Queen's College of Physicians, on the 18th of May last. They were examined with great interest by the members and visitors present, and the degree of vision which one of them evidently possessed, reflects the highest credit on the ingenuity, patience, and manual dexterity of the scientific operator. The results of these eighteen experiments were: in ten, the iris was injured; in eleven, the crystalline lens escaped; in seventeen, union took place between the implanted cornea and the adjacent surfaces in forty-eight hours, so as to admit of the withdrawal of the ligatures, which are always a great source of irritation; in four, three ligatures were employed; in fourteen, only two, and with equally favorable results; in twelve, adhesion of the iris to some part of the cicatrix ensued; in one, sloughing of the cornea and destruction of the eye took place, an event which arose from the cornea being kept for half an hour without applying it, with the view of ascertaining how long it would be likely to retain a sufficient degree of vitality to enable it to unite. Of the whole eighteen experimented on, sixteen recovered imperfect vision. The difficulty of performing the experiment in such a way as to afford a chance of preserving the transparency of the implanted cornea, was a source of much disappointment to Dr. Bigger, and for a long period he could not succeed in devising any means for this purpose, until after his eighth experiment at home, when he discovered that much benefit might be derived from the local application of bichloride of mercury. A weak solution of this salt, gradually increased to the extent of three grains to the ounce of distilled water, and dropped into the eye three or four times a day, after the cornea had become adherent, was found by him to exercise an almost specific action in diminishing the opacity of the implanted cornea."

3. "With reference to the applicability of the operation to the human species, Dr. Bigger observed, that he thought that in man the chances of success would be greater, at least so far as steadiness during the operation, avoidance of injury, and other obvious circumstances might contribute to that desirable end. With respect to the animal from which the cornea would be taken in the case of the human subject, Dr. Bigger has not yet decided, and invites the attention of comparative anatomists to this point of the investigation. The animal whose cornea he has found to make the nearest approach to that of man is the pig; it is, however, much thicker and coarser in its texture. In a spirit of just and humane feeling, he deprecates the removal of the cornea from the human eye, even when permitted for gain by the possessor; but thinks that a person afflicted with incurable amaurosis might be prevailed on to part with his pellucid cornea, which might be replaced by one taken from some of the inferior animals. He thinks, however, that the operation should not be sanctioned under any circumstances, when the patient enjoys even a tolerable degree of vision with the other eye, at least until our knowledge has been increased by further experiments and observations. He is of opinion that cases of blindness caused by small-pox, ulcers on the cornea, and ophthalmia not affecting the deeper structures of the eye, would be the most favorable for operation."

*Dublin Journal. July,* 1837.

---

*Observations on the Advantage of Healing by the first intention the Wound in the Lateral Operation for Lithotomy.* By John Chrichton, Esq., Surgeon, Dundee.

The object of this communication is expressed in its title; and it contains a report of many interesting facts confirming the views of its highly respectable and skilful author. In a former communication to the same Journal, in the year 1828, Mr. Chrichton stated, that he "had already witnessed the wound healed by the first intention in five instances, the urine passing from the urethra from the commencement, and continuing to do so, whereby the cases were remarkably expedited, and much annoyance from the dribbling of the urine by the wound avoided." Since that time about forty cases have been operated on by Mr. C., and in thirteen of these "the wound adhered at once, healing by the first intention. In the greater number, however, the chylopoietic viscera being unsound, and the habit much debilitated, the attempt to heal the wound by the first intention was not considered advisable." Mr. C. concludes his paper with some severe, and we must be permitted to say, rather partial strictures on the operation of lithotrity. We recommend to his attention the very admirable and most candid essay of Mr. Key on this subject, noticed in our present Number.

*Edinburgh Journal. July,* 1837.

---

*Case of Club Foot successfully treated by the Division of the Tendo Achillis.* By G. Ray, M.R.C.S.

This is a very interesting and well-detailed case, and strikingly illustrative of the benefit resulting from the plan of dividing the tendon in such cases. The patient was Mr. Ray's own son, aged fourteen years, and the deformity was congenital.

"On the 1st of May, Dr. Little divided the tendo-achillis, about an inch and a half above the malleolus, by introducing a very narrow, slightly-curved, bistoury between the tendon and the deeper-seated muscles and tibial vessels, directing the edge of the knife against the front of the tendon, dividing it from within outwards, leaving the skin covering the tendon untouched. The section was accomplished most skilfully, and the *minute* puncture in the skin was covered with a strip of adhesive plaster, and a loose bandage was applied round the foot and leg. Any separation of the divided ends of the tendon was prevented by placing the foot in its deformed position upon a stiff pasteboard splint, applied along the outer side, and by adjusting the limb, with the knee bent, upon a pillow; on the second day the report was that he had had no pain or tenderness, and would not have known that anything had been done to the limb but for occasional contractions of the muscles of the calf, they having lost their fixed point. 3d day. The puncture (which is inconceivably small) was found agglutinated, although not firmly cicatrized; the application of the extension foot-board was consequently deferred another day or two. The ends of the divided tendon cannot be felt, owing to the effusion of coagulable lymph, which is necessary for its perfect reunion. 4th day. The puncture firmly cicatrized, and Stromeyer's foot-board applied. The boy is perfectly well. The handling of the foot preparatory to the application of the apparatus, and the attempt to draw the heel down, produce no pain. 10th day. The stretching of the lymph effused between the ends of the tendo-achillis, the elongation of the contracted ligaments of the anklejoint, and the consequent bending of the foot upon the leg, have been gradually continued, so that the foot at present is nearly at a right angle with the leg, and all this has been effected without pain. He has been able for the last two or three days to walk about the room, with the foot-board, which diminishes the irksomeness of wearing it, and assists much in bringing down the heel to the ground.

"14th day. The foot is completely at a right angle with the leg."

At the end of six weeks the boy returned home, and is stated to be steadily improving; the lameness being very slight and becoming less daily.

*Lancet. July* 15, 1837.

---

*On the Treatment of Gonorrhœa in the Female by solid Nitras Argenti.*
By Joseph Bell, Esq.

We notice this communication for the purpose of obviating the effect of the statement made by Dr. Smith, and recorded in our last Number, p. 259. The following extract from Mr. Bell's paper sufficiently disproves the most important of Mr. Smith's charges:

"Mr. Smith deems the practice in question 'cruel.' In support of this surmise, he states that the 'Lock Hospital is always full when Dr. Cumin has charge of it; but, on the other hand, does not average above four-fifths full when under the charge of Dr. Hannay.' * * *

"The following statement, made from the journal of the Lock Hospital, will, however, set this matter in its true light:

*Number of Patients in the Hospital during Dr. Cumin's six months' attendance, commencing 1st January,* 1836, *ending 1st July,* 1836.

| | |
|---|---|
| Number of patients remaining in the hospital on 1st January . . | 24 |
| Number of ditto admitted from 1st of January to 1st of July . . | 135 |
| Total number of ditto under Dr. C.'s charge . . . . . . | 159 |
| Of these there remained in the hospital on 1st July . . . . . | 25 |
| Number of patients dismissed under Dr. C. . . . . . . | 134 |

*Number of Patients in the Hospital during Dr. Hannay's six months' attendance, commencing 1st July,* 1836, *and ending 1st January,* 1837.

| | |
|---|---|
| Number of patients remaining in the hospital on 1st July . . . | 25 |
| Number of ditto admitted from 1st July, 1836, to 1st January, 1837 . | 130 |
| Total number of ditto under Dr. H.'s charge . . . . . . | 155 |
| Of these there remained in the hospital on 1st January, 1837 . . | 15 |
| Number of patients dismissed during Dr. H.'s attendance . . . | 143. |

*Medical Gazette. June* 24, 1837.

